# Tiny Actors in the Big Cellular World: Extracellular Vesicles Playing Critical Roles in Cancer

**DOI:** 10.3390/ijms21207688

**Published:** 2020-10-17

**Authors:** Ancuta Jurj, Cecilia Pop-Bica, Ondrej Slaby, Cristina D. Ştefan, William C. Cho, Schuyler S. Korban, Ioana Berindan-Neagoe

**Affiliations:** 1 Research Center for Functional Genomics, Biomedicine and Translational Medicine, “Iuliu Hațieganu” University of Medicine and Pharmacy, 400337 Cluj-Napoca, Romania; ancajurj15@gmail.com (A.J.); cecilia.bica8@gmail.com (C.P.-B.); 2 Central European Institute of Technology, Masaryk University, 625 00 Brno, Czech Republic; on.slaby@gmail.com; 3 Department of Pathology, Faculty Hospital Brno and Faculty of Medicine, Masaryk University, 625 00 Brno, Czech Republic; 4 SingHealth Duke-NUS Global Health Institute, Singapore 169857, Singapore; cristinastefan10@gmail.com; 5 Department of Clinical Oncology, Queen Elizabeth Hospital, Hong Kong, China; chocs@ha.org.hk; 6 Department of Natural Resources and Environmental Sciences, University of Illinois at Urbana-Champaign, Urbana, IL 61801, USA; korban@illinois.edu; 7 Department of Functional Genomics and Experimental Pathology, “Prof. Dr. Ion Chiricuta” Oncology Institute, 400015 Cluj-Napoca, Romania

**Keywords:** extracellular vesicles, cancer, therapeutic agents, cell-to-cell communication

## Abstract

Communications among cells can be achieved either via direct interactions or via secretion of soluble factors. The emergence of extracellular vesicles (EVs) as entities that play key roles in cell-to-cell communication offer opportunities in exploring their features for use in therapeutics; i.e., management and treatment of various pathologies, such as those used for cancer. The potential use of EVs as therapeutic agents is attributed not only for their cell membrane-bound components, but also for their cargos, mostly bioactive molecules, wherein the former regulate interactions with a recipient cell while the latter trigger cellular functions/molecular mechanisms of a recipient cell. In this article, we highlight the involvement of EVs in hallmarks of a cancer cell, particularly focusing on those molecular processes that are influenced by EV cargos. Moreover, we explored the roles of RNA species and proteins carried by EVs in eliciting drug resistance phenotypes. Interestingly, engineered EVs have been investigated and proposed as therapeutic agents in various in vivo and in vitro studies, as well as in several clinical trials.

## 1. Introduction

Both solid and hematological malignant tumors are not isolated entities. In fact, they involve complex systemic networks involving cell-to-cell communications between tumor cells and accompanying modified cells. Moreover, both tumor progression and invasion are sustained by a complex microenvironment. This is comprised of networks of components, including cancer-associated fibroblasts, endothelial cells, lymphocytes, and macrophages, as well as secreted factors and elements of the extracellular matrix. Interactions among neighboring cells through a direct cell–cell contact is essential for tumor growth and development, while intercellular communication provides a complex system of secreted factors [1].

To manage all components present in multicellular organisms, cellular communication is critical. McCrea et al. wrote an inspirational quote on the intercellular communication “the music that the nucleus hears” [2]. Communication involves sharing of information through several signaling mechanisms that are either direct (intracrine/autocrine and juxtacrine) and/or indirect (endocrine, paracrine, and synaptic) communications [3]. In this regard, all types of cells have been shown to release and receive both soluble factors and membrane-derived vesicles, the latter receiving increasing attention in the past decades [4]. The first instance of the presence of membrane-derived vesicles is observed in reticulocytes, wherein released vesicles would remove transferrin receptors from the cell, an important step in their maturation to erythrocytes [5]. Early on, these membrane-derived vesicles have been initially deemed as cellular “garbage bags”. Subsequently, numerous studies have been undertaken to investigate membrane-derived vesicles detected on primary cells [6]; i.e., primary cells of the immune and nervous systems, and cancer cell lines [7]. It has been reported that extracellular vesicles (EVs) can be isolated from various bodily fluids, as they play important roles in the management of various normal physiological processes, including stem cell maintenance [8], immune surveillance [9], tissue repair [10], and blood coagulation [4].

It is reported that physical and molecular characteristics of EVs have impacts on various biological processes, including cancer development, progression, and metastasis [11]. Moreover, small sizes of EVs offer critical properties, including immune system escape, biocompatibility, and biodegradability, as well as transfer of their contents into both neighboring and distant cells. During biogenesis, EVs acquire important bioactive molecules that regulate several biological processes. Thus, cancer-derived EVs have been largely described as possessing both pro- and antitumor functions. For example, tumor-derived EVs interact with immune cells by delivering negative signals and interfering with their antitumor functions. By suppressing immune cell functions, EVs promote cancer progression and facilitate tumor escape. Moreover, EVs carry important molecules and factors that either directly or indirectly influence several processes, including development and maturation, as well as antitumor activities in immune cells [11]. Conversely, antitumor effects of EVs have been observed in dendritic cell-derived EVs, and these are capable of being used in immunotherapy [12].

It has been observed that EVs are tightly linked to tumorigenesis [13], spread of pathogenic agents and viruses (e.g., the Human Immunodeficiency Virus-1 [HIV-1]), amyloid-β-derived peptides [14], and α-synuclein [15] (linked to Alzheimer’s and Parkinson’s diseases). Due to varied compositions of EVs, they have been deemed useful in the fields of both diagnostics and therapeutics [16]. Moreover, EVs can be potentially useful in serving as drug delivery vehicles by transporting several molecular species as part of normal cell-to-cell communication.

In this review, we will discuss the potential and role(s) of EVs in modulating both physiological and pathological processes, as well as how these entities can be used as therapeutic agents [17].

## 2. The War Waged Inside the Cell

EVs are described based on their size, cellular origin (endosome- or plasma membrane-derived), biological function, and biogenesis process. Moreover, when described based on their biogenesis, EVs are cataloged into apoptotic bodies, microvesicles, and exosomes [18]. These major classes are cell-based vesicles having diameters ranging between 30 and 2000 nm (Table 1). Furthermore, these entities exhibit different properties that help distinguish them among all main classes of EVs. Differences among different EV classes are based on the content, size, route of biogenesis, and surface markers [19].

One of the largest cell-based vesicles is those of apoptotic bodies that are released by any type of cell once apoptotic processes are activated. Specifically, apoptotic EVs are generated during plasma membrane blebbing during apoptosis, as these are phagocytosed by macrophages and then fused with lysosomes [20]. Generally, these EVs are known to carry nuclear fragments and cellular organelles as a result of cell fragmentation [20]. Furthermore, these EVs are characterized by a flip of phosphatidylserine along an external layer, a permeable membrane, and expression of phagocytosis-promoting signals (calreticulin [21] and calnexin [22]), as well as chemokines and adhesion molecules, including ICAM3 and CX3CL1/fractalkine, and MHC class II molecules [23]. These are all important for direct antigen presentation CD4+ T cells and immunological memory activation [23].

Microvesicles, also known as ectosomes, are usually larger than 0.2 µm in size, and they are released outward from the plasma membrane via budding or shedding into the extracellular matrix. The process of microvesicle formation is mediated through a complex process involving cytoskeletal protein contraction and phospholipid redistribution [24]. During biogenesis, microvesicles are mainly composed of a plasma membrane and of cytosolic-associated proteins [19]. Microvesicles are involved in several key functions, including intercellular communication, signal transduction, and immune regulation. In particular, these entities mediate tumor invasion, inflammation, metastases, stem-cell renewal, and expansion [25]. During biogenesis, microvesicles receive important structural components, including Flotillin-2, Annexin V, integrins, selectin, CD40, and metalloproteinase [26].

**Figure 1:**
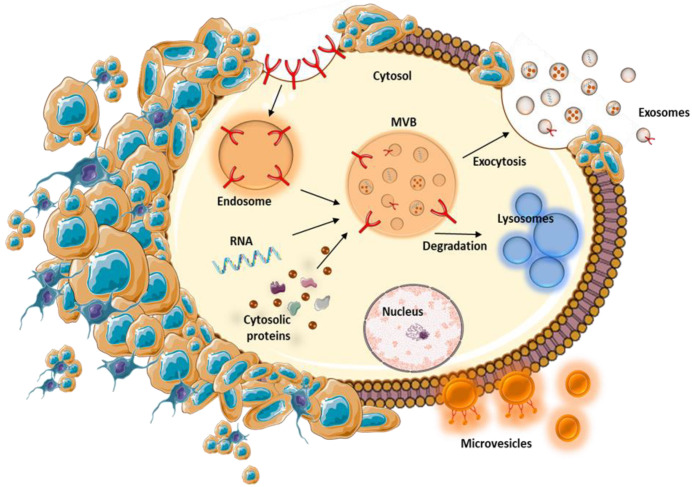
Biogenesis mechanisms of EVs, exosomes, and microvesicles. Endocytosis, an active process, begins with the generation of endosomes after cells are internalized within the extracellular fluid material to form internal vesicles and early and late endosomes. Furthermore, multivesicular bodies (MVBs) are formed via inward budding of a late endosomal membrane. Moreover, MVBs can fuse with either the plasmalemma, releasing their cargo into extracellular space, or with lysosomes, wherein their contents are degraded.

In contrast, exosomes are between 30–100 nm in size, and are generated using the endosomal pathway [25]. Exosome biogenesis begins with the formation of early endosomes that undergo inward (or reverse) budding and then subsequent formation of intraluminal vesicles (ILVs), and referred to as multivesicular bodies (MVBs) or late endosomes. As a final step, late endosomes may either directly fuse with lysosomes, wherein the endocytosed cargo is degraded, or they may fuse with the plasmalemma releasing its ILVs (exosomes) to the extracellular space [25,27]. ESCRT (endosomal sorting complexes required for transport) is a molecular complex that plays an important role in MVB formation and regulation (Figure 1). Specifically, ESCRT is formed from the other four molecular complexes, including ESCRT-0, -I, -II, and -III. These multi-protein complexes are responsible for different functions, depending on their components. ESCRT-0 is dependent on ubiquitin and determines clustering of the cargo, ESCRT-I and ESCRT-II play important roles in bud formation, and ESCRT-III determines scission of vesicles. In addition, accessory proteins (VPS4 ATPase) are implicated in the final steps of ESCRT functions, namely of dissociation and recycling. In many studies, other ESCRT-independent pathways of MVB formation have been observed [28]. Some classes of molecules implicated in ESCRT-independent mechanisms of exosome biogenesis are represented by proteolipid proteins, tetraspanins, and heat shock proteins [29].

In general, following MVB fusion with the plasmalemma, exosomes are secreted from cells. This mechanism is regulated via two mechanisms, constitutive and inducible. The constitutive mechanism is managed by a plethora of molecules, including heterotrimeric G-proteins, flotillins, and glycosphingolipids [30], while inducible secretion is determined by stress stimuli, including thrombin, DNA damage, hypoxia, heat shock, and lipopolysaccharide (LPS) stimulation [27].

**Figure 2:**
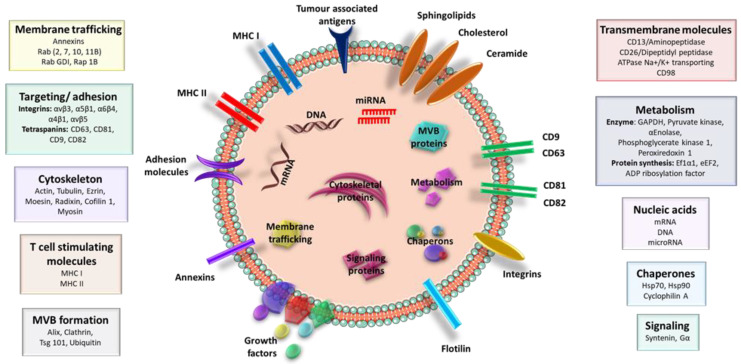
EVs’ cargo profile. EVs are secreted by a wide range of cells, normal and tumor, having the capacity to deliver various bioactive molecules including nucleic acids, specific proteins, and lipids from the donor cells to recipient cells

During biogenesis, exosomes receive critical bioactive molecules from donor cells, including nucleic acids, lipids, and proteins, that are specific for each cell type [40]. For composition of both exosomes and microvesicles, the following components are important: mRNAs, microRNAs (miR), non-coding RNAs, DNAs (mtDNA, ssDNA, and dsDNA), mRNA cytoplasmic proteins, and lipid raft-interacting proteins (Figure 2) [41]. Recent attention has focused on understanding how DNAs are packaged within EVs. In this regard, several research groups have reported on the presence of DNAs (mtDNA, ssDNA, and dsDNA) in EVs secreted from various types of malignancies, including melanoma, breast, lung, pancreas, and prostate cancer [42]. However, there is little knowledge of the origin, biological significance, and mechanism of DNA packaging in EVs. Conversely, few studies have reported that DNA is located along the outer surface and not within EVs [43,44]. Thus, it is proposed that outer surfaces of EVs are capable of interacting with proteins, nucleic acids, and other molecules regulating motility, aggregation, and various other important processes for EVs [45]. Furthermore, cargos within these vesicles can influence recipient cells [46], thus suggesting that exchanges of EV cargos between either normal or cancer cells may represent an effective and efficient intercellular communication when cells have particular physiological behaviors, but these are dramatically altered in cancer cells. Alongside nucleic acids, exosomal proteins are specific, and they are present in endocytic compartments of donor cell membranes, as well as in cellular membranes, the nucleus, the cytosol, and the Golgi apparatus, as well as in the endoplasmic reticulum and mitochondria, but at lower frequencies for these latter two organelles [47]. Tetraspanins (CD9, CD63, CD81, and CD82) are among some of the most typical proteins present in exosomes, alongside GPI-anchored proteins and receptors. Moreover, within interiors of exosomes, several molecular species of a parent cell are encased, and these are represented by structural components, heat shock proteins, chaperones, and enzymes involved in metabolic processes, among many others (Figure 2) [17,27].

Interestingly, EVs are carriers of essential soluble immune mediators, including cytokines and chemokines. Several cytokines, such as IL-1α, IL-1β, IL-6, IL18, and IL-32, are engulfed within EVs. In endothelial cell-derived apoptotic bodies, IL-1α is present; whereas, IL-18 is associated with EVs shed from surfaces of macrophages. Additionally, IL-6 and IL-32 are secreted by mast cells upon IL-1 stimulation [48]. Moreover, heat-stressed tumor cells have been shown to release EVs with different CCL compositions compared to their nonstressed counterpart [49].

## 3. EVs Isolation and Characterization

EVs can be isolated from different biological fluids (plasma, serum, saliva, milk, and urine, among others), as well as from cell culture supernatants. There are several available methodologies to remove undesirable particles from samples of interest. In cell cultures, EVs are separated from other components of cell media using differential centrifugation. This technique utilizes centrifugal force to separate contaminants from EVs, along with several necessary steps to remove cells, cell debris, and large microvesicles in order to obtain purified EVs [50]. Another isolation technique, density gradient centrifugation, separates EVs into specific layers in different solutions (sucrose, iohexol, and iodixanol) depending on their buoyant densities [51]. In this method, subcellular components, including mitochondria, endosomes, and peroxisomes, are successfully separated into distinct layers within the density gradient solution [52]. In yet another method, size-exclusion chromatography utilizes porous beads to separate biomolecules based on their hydrodynamic radii [53]; thus, biological samples are filtered through a column of porous beads of radii smaller than those of EVs [54]. Similarly, filter-based enrichment methods also depend on the sizes of EVs for separation, but instead of porous beads, sieves are used. Further, antibody enrichment methods are based on selecting for markers specific for EVs, such as CD9, CD63, and CD81, thus serving as complementary to size-based methods, thereby capable of specific selection of EVs [55]. Recently, acoustics and/or microfluidics methods have been developed that will isolate EVs in label-free and contact-free manners [56,57]. In addition, EVs can be separated from biological samples via precipitation using different chemicals, such as polyethylene glycol (PEG), sodium acetate, or protamine. It has been reported that using PEG, both EVs and proteins are precipitated into a pellet that can be further analyzed [58]. Similarly, magnetic beads coated with antibodies for common EV surface proteins (CD9, CD63, and CD81) are used [59]; whereas, a fluidic technique, ExoTIC (exosome total isolation kit), utilizes step-wise nanoporous membranes to trap molecules or particles of specific sizes, thereby allowing for smaller molecules and particles to flow through a membrane filter [60]. This latter method may be deemed as the most accurate size-based method used to isolate EVs from biological samples with a high yield of intact EV structures.

As EVs, of nano-sizes, must be quantified and evaluated for purity, there are several methods that can determine the numbers of vesicles released and cell type (detection of surface antigens), as well as EV morphological traits [61]. Dynamic light scattering (DLS) is based on a particle’s Brownian motion in solution, used to measure the size distribution of particles, as well as their zeta potentials, measuring diameters of particles ranging between 1 nm and 6 µm [62]. However, this technique does not provide any biochemical data of purified EVs [62]. In another technique similar to DLS, nanoparticle tracking analysis (NTA) is used to measure concentration, count, and size distribution of EVs based on their Brownian motion; moreover, this technique can measure smaller-sized EVs, ranging from 1 to 1000 nm [63]. In yet another technique, flow cytometry is used to indirectly quantify EVs as it is based on using specific antibodies that accurately recognize EV markers from a liquid medium. However, flow cytometry cannot evaluate the complex profiles of subsets of EVs. Similar to DLS and NTA, flow cytometry is capable of providing data on EV size, count, and distribution [64]. Finally, both EV purity and quality can be determined using transmission electron microscopy (TEM) wherein standard traits, such as cup-like structures and lipid bilayers, can be determined [65]; whereas, EV purity can be assessed based on presence or absence of protein markers [50].

## 4. Biological Roles of EVs

EVs, particularly exosomes, play important roles in cells by influencing several biological processes. Their effects on receptor cells can be exerted via various mechanisms, such as phagocytosis, direct receptor binding, and receptor-dependent internalization. Thus, EVs can deliver information through a wide range of mechanisms, thereby playing important roles in tissue repair [10], stem cell maintenance [8], and immune surveillance [9]. Due to their pleiotropic actions, EVs have been, time and time again, deemed as signalomes.

It has been reported that EVs can influence activities of immune cells present both in the tumor microenvironment and in the circulatory system [66]. Once EVs are internalized into targeted cells, they release their cargo and exert their role by activating different biological mechanisms. EVs can mediate the activation of immune cells by promoting proliferation and survival of hematopoietic stem cells, as well as activation of monocytes [67], B lymphocytes [66], and NK cells [68]. EVs can also inhibit immune responses via regulation of NK and CD8^+^ cell activities [69] and activation of Treg cells, as well as inhibition of dendritic cell (DC) maturation [70] and formation [71]. For those EVs derived from stem cells, they have been demonstrated to regulate stem cell maintenance with implications in tissue regeneration [72]. In addition, it has also been shown that EVs can modify stem cells to develop into either a liver cell phenotype [73] or a lung phenotype [74].

## 5. Pathological Roles of EVs

**Figure 3:**
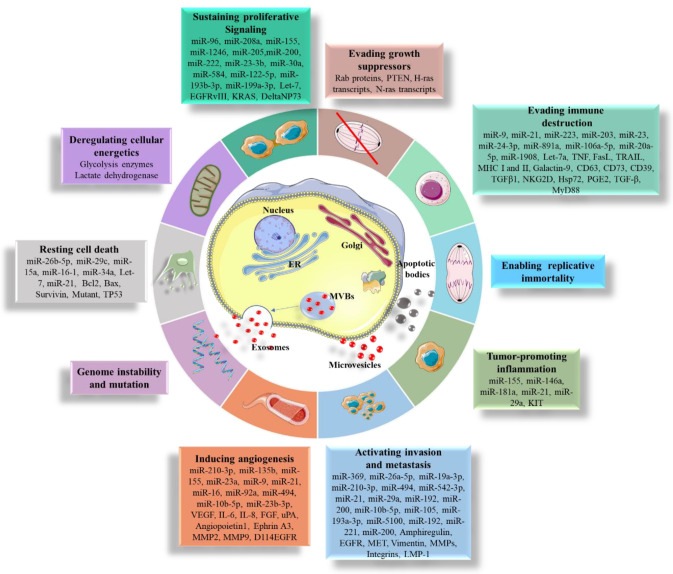
A schematic representation of the impact of tumor-derived EVs on the hallmarks of cancer. Pro-oncogenic molecules can be transported through the cellular membrane by EVs and microvesicles. Molecules transported via EVs have been reported to contribute to each of the hallmarks of cancer. Abbreviations: ER, endoplasmic reticulum; MVBs, multivesicular bodies

It is important to point out that EVs can be secreted by malignant or deregulated cells. During biogenesis processes, EVs are loaded with important bioactive molecules from malignant cells that influence the phenotype(s) of target cells. It has been reported that EVs are implicated in the formation of a premetastatic milieu throughout the body [75]. Moreover, EVs are also involved in other critical biological processes and have the capability of stimulating tumor progression [13]. This process is sustained by EVs via delivery and release of their targets into a target cell(s). Alongside tumor progression, EVs have the capability of carrying out other critical processes, including cell proliferation, tumor growth [76], angiogenesis [77,78,79,80,81,82,83,84,85], matrix remodeling, metastasis [75,86,87,88,89,90,91,92,93,94,95,96], immune escape [69,97,98,99,100,101,102,103,104,105,106,107,108,109], resistance to apoptosis [110,111,112,113], deregulation of energetic metabolism [114,115,116,117], sustaining proliferative signaling [94,118,119,120], evading growth suppression [121,122,123], deregulating and tumor-promoting inflammation [100,124,125] (Figure 3).

### 5.1. Promoting Cell Proliferation and Resistance to Apoptosis

EV transfer can modify particular signaling pathways in the target cell, modifying proliferation and resistance to apoptosis, among other processes. For example, it has been reported that in gastric cancer, cell proliferation can be enhanced through exosomal transfer of CD97 that activates the Mitogen-Activated Protein Kinase (MAPK) pathway [126]. In chronic myeloid leukemia, it has been observed that cellular proliferation is promoted via induction of phosphatidylinositol 3-kinase (PI3K)/protein kinase B (AKT) and MAPK pathways [127]. For instance, melanoma-derived EVs transfer PDGFR-8, which in turn activates the PI3K/AKT pathway in target cells [128]. Moreover, PI3K/AKT and MAPK pathways are reported to be activated in both gastric and bladder carcinomas by EVs [129]. In addition, EVs derived from glioblastoma are reported to promote cell proliferation in a CLIC1-dependent manner [130]. Soekmadji et al. have demonstrated that EVs derived from prostate cancer cells cultured in the presence of androgens are enriched in CD9, which promotes proliferation of androgen-deprived cells [131]; whereas, Matsumoto et al. have reported that mice injected with melanoma-derived EVs result in accelerated in vivo growth of murine melanomas [132].

EVs can also alter target cell(s) via their miR content as it has been shown that miR-93-5 from esophageal cancer-derived EVs inhibits phosphatase and tensin protein (PTEN) expression stimulating cell proliferation [133]. Other important examples of EVs’ role in stimulating cell proliferation has been reported in colon cancer wherein EVs carry higher levels of miR-200b and miR-193a [134] and of pancreatic cancer-derived EVs loaded with miR-23b-3p and of papillary thyroid cancer-derived EVs loaded with miR-222 [135]. Furthermore, it has been observed that tumor-derived EVs actively transfer miR-106a-5p, miR-891a, miR-24-3p, and miR-20a-5p that promote cell proliferation via alteration of Microtubule Affinity-Regulating Kinase1 (MARK1) signaling in human nasopharynx cancer [136]. Moreover, EV miR-302b is delivered from lung carcinoma cell lines to target cells, leading to cell growth inhibition via the TGFβRII/ERK signaling pathway [137], while EV miR-584 accelerates cell proliferation in hepatocellular cancer cells [138].

### 5.2. Promoting Cell Migration

In addition to their effects on cell proliferation, EVs secreted by tumor cells can also alter the migratory status of malignant cells. EVs derived from nasopharyngeal carcinoma carrying epithelial-mesenchymal transition (EMT)-inducing signals, including HIF1α, TGFβ [98], and matrix metalloproteinases (MMPs), were reported to improve the migratory capacity of tumor cells [139]. Interestingly, EVs from a hypoxic prostate cell line have been shown to lead to increased mobility and invasiveness in a naïve human prostate cancer cells [140]. Moreover, EVs secreted from muscle-invasive bladder cancer contributed to decreased levels of E-cadherin as well as to enhanced migration and invasion in uroepithelial cells [141,142]. In another study, EV miR-105 was reported to stimulate invasion in both the respiratory and central nervous systems by inhibiting ZO-1 in endothelial cells, leading to enhanced cell migration [143]. Furthermore, it has been observed that EV miR-21 stimulates invasion of esophageal tumor cells by activating the programmed cell death 4 (PDCD4)/ c-Jun NH_2_-terminal kinase (JNK) axis [90].

### 5.3. Sustaining Angiogenesis

It has been reported that induction of a mutated epidermal growth factor receptor variant III (EGFRvIII) in glioma cells would lead to increased vesiculation and transfer of the mutated EGFRvIII to other cells and to increased vascular endothelial growth factor (VEGF) production [94]. In addition, it has been observed that EVs from primary glioblastoma cells are loaded with miRs that influence angiogenesis [76]. Recently, it has been demonstrated that EGFR can be transferred to endothelial cells wherein expression of VEGF is induced along with subsequent autocrine activation of VEGF-R2 [77]. Thus, these findings suggest that EVs can result in tumor growth by stimulating cancer cell proliferation and activating angiogenesis in adjacent endothelial cells [77]. Kim et al. have reported that sphingomyelin expressed on tumor cells-derived EVs stimulate processes, such as migration and angiogenesis, in endothelial cells [144]. It has been observed that such EVs secreted by tumor cells are enriched in MMPs as well as in CD147. These components have been proposed to play roles in both hydrolysis of the extracellular matrix and initiation of angiogenesis [145]. Interestingly, it has also been observed that pSTAT5 can be transferred to endothelial cells via EVs, and that it is capable of activating ERK1/2 along with subsequent angiogenesis stimulation [146]. Moreover, miR-214 is also responsible for promoting angiogenesis by suppressing Ataxia Telangiectasia Mutated (ATM) expression and preventing senescence [147]. In fact, mesenchymal stem cells-derived EVs have also been shown to stimulate the angiogenesis process, as demonstrated in vivo in an ischemic heart model [148].

Colon cancer cells have been shown to transfer miR-25-3p to endothelial cells not only by stimulating angiogenesis but also by increasing vascular permeability [149]. EVs secreted by hepatocellular carcinoma cells have been shown to transfer miR-103 to endothelial cells, leading to a reduction in the integrity of endothelial junctions, and thereby increasing vascular permeability [150]. The angiogenesis process has also been shown to be stimulated by miR-145-5p and miR-14-3p from lung cancer-derived EVs [151]. Moreover, in lung cancer cells, release of EV miR-21 stimulates angiogenesis in nontumor lung cells [90]. In another study, miR-9 exhibits proangiogenic activity by reducing expression levels of the *SOCS5* gene and by promoting Janus kinase/signal transducers and activators of transcription (JAK-STAT) signaling, thereby supporting migration of endothelial cells and tumor angiogenesis [152]. Furthermore, increased expression levels of EV miR-9 can differentiate an osteoblast precursor cell line into osteoblast cells and upregulate angiogenesis via an AMPK-dependent pathway [153].

From a therapeutic perspective, it has been observed that EVs can be used to shed bevacizumab, an anti-VEGF antibody, thus leading to decreased efficacy in glioblastoma [154]. Additionally, some cancers are capable of secreting VEGF isoforms with reduced affinities for bevacizumab, leading to another therapy escape mechanism [155]. Another antiangiogenic agent commonly used throughout the field of oncology is sorafenib. Hepatocellular carcinoma-derived EVs have been shown to activate the HGF/MET/AKT pathway in sensitive hepatocellular carcinoma cells, thereby inducing sorafenib resistance. Moreover, it has been observed that more invasive cell lines are capable of better inducing sorafenib resistance compared to less invasive cell lines, thus demonstrating that different malignant subclones are capable of sharing their acquired resistance [156].

It has been reported that sorafenib induces increased expression of linc-ROR in EVs secreted by hepatocellular carcinoma cells [157]. EVs have also been shown to transfer resistance to sunitinib, a similar compound to sorafenib, to hepatocellular carcinoma subclones [157], as well as to different subclones of renal cell carcinoma [158].

### 5.4. Immune System Evasion

One of the important functions of the immune system is to recognize and to destroy particular cells that present alterations when compared to self-antigens of unaltered (normal) cells. However, this function can be evaded by malignant cells either by changing surface antigens of malignant cells or by influencing the immune system. The role(s) of EVs in this process has been reported in various studies [80]. It has been demonstrated that EVs secreted from tumor-derived macrophages are enriched with particular miRs that enhance the local invasion of breast cancer cells [103]. In fact, the effects induced by EVs are related to modulation of the immune response. Furthermore, it has been demonstrated that EVs of tumor cells are capable of promoting immune escape by determining regulatory T cell expansion [159] and by shedding FAS ligand (FASL), as well as by inducing CD8^+^T cell apoptosis and increasing expression of the *MMP9* gene in melanoma cells [79,160].

Recently, it has been reported that EVs can express PD-L1, thus suppressing activities of antitumor T-cells [161]. Moreover, it has been observed that EV PD-L1 expression is inversely correlated with nivolumab and pembrolizumab response [162]. These findings are of particular importance in checkpoint blockade therapy as this reveals that EVs can act as decoys for therapeutic agents. As checkpoint blockers, this would allow for adjustment of the dosage of therapy by taking into consideration EV expression of particular markers, such as PD-L1. In other cancers, such as head and neck squamous cell carcinoma, it has been observed that there are differences between EV cargos in patients experiencing relapse compared to those who remain in remission at two years following ipilimumab therapy [163]. More specifically, it has been observed that for patients in remission, at two years, have lower numbers of EVs positive for both CD3 and CTLA4. Conversely, it has been shown that patients who relapsed after two years have increased numbers of EVs derived from Treg cells, thus demonstrating the importance of EVs in mirroring the T-cell response to tumor cells [163].

Immunomodulatory effects of EVs have also been reported in gastric cancer [164]. It has been observed that EVs isolated from gastroepiploic veins have shown increased levels of TGF-β1 expression for patients presenting either lymph nodes or distant metastasis. This finding has demonstrated the role of EVs in preparing an immunosuppressive premetastatic niche for engraftment of circulating tumor cells [164]. Although not explored in the abovementioned study, it is likely that checkpoint inhibitors could reverse these observed generated immunosuppressive premetastatic niches along with reduced probability of gastric cancer reaching advanced stages.

In other studies, it has been observed that EV miR-212-3p from pancreatic cancer cells have degraded RFXAP mRNAs in dendritic cells (DCs), leading to immune tolerance by minimizing expression of MHC II [165]. Furthermore, hypoxic tumor cells-derived EVs influence functions of natural killer (NK) cells by delivering miR-23a and TGFβ [166], while miR-214 secreted from human embryonic kidney cells induces immunological tolerance responses in CD4+ T-cells [167].

### 5.5. Transferring Mutations

Tumor-derived EVs have DNA fragments that can be transferred to recipient cells [45]. It has been reported that resistant melanoma cells can activate the MAPK pathway in sensitive melanoma cells through an EV-mediated truncated ALK transfer [168]. Moreover, EVs positive for EGFRvIII have been shown to activate both MAPK and PI3K/AKT pathways [94]; whereas, β-catenin-mutated colon cancer cells are reported to transfer their mutation to β-catenin wild-type cells along with subsequent activation of the β-catenin/WNT pathway [169]. In addition, a mutated SMAD4 is observed to be transferred from resistant to sensitive ovarian cancer cells, leading to an increased platinum resistance [80].

## 6. EVs in Cancer Stem Cells

As EVs play important roles in cancer cells, it is known that particular subpopulation(s) within a malignant mass, cancer stem cells (CSCs), present significant chemoresistance and are generally deemed as seeds for relapse [170]. EVs derived from CSCs are reported to transfer particular information to other cells. For example, EVs derived from renal cell carcinoma stem cells have been shown to carry a specific miR signature that influences levels of PTEN in target cells. This change is functionally translated into increased EMT followed by a subsequent increase in frequency of metastasis [88,171].

EVs derived from glioblastoma stem cells contain miR-21, which can be transferred to endothelial cells, leading to upregulation of angiogenesis via the miR-21/VEGF pathway [172]. In another study, macrophages treated with glioblastoma cancer stem cell-derived EVs can skew macrophages to an anti-inflammatory phenotype (M2), associated with increased expression of PD-L1 on surfaces of these cells, thus demonstrating immunosuppressive roles of these EVs [173]. On the other hand, EVs from thyroid CSC spheroids can induce a stem cell-like phenotype in recipient cells by increasing levels of SOX2. Moreover, it has been shown that EVs derived from these cells also increase the EMT through SLUG upregulation [174].

EVs from CSCs have also been shown to influence the immune system, as EVs derived from colorectal CSC are reported to increase IL-1β in neutrophils, thereby inducing a pro-inflammatory environment [175].

## 7. EVs in Drug Resistance

One of the most heavily investigated characteristics of EVs is their ability to transfer resistance to particular therapeutic compounds. This is due to their capability of transferring specific molecular traits, such as efflux pumps or pathway regulation, thus rendering a phenotype better adapted to a particular selected therapeutic strategy [80]. Often, efflux pumps are transferred from resistant to sensitive cells [176,177,178,179,180]. These efflux pumps induce tumor resistance, corresponding to the transfer of ATP-binding cassette (ABC) family members, of which the multidrug resistance 1 (MDR1) and multidrug resistance-associated protein 1 (MRP1) have attracted attention in oncology [176,177,178,179,180]. More specifically, MRP1 can be transferred from resistant acute promyelocytic leukemia to sensitive cells [176]. Additionally, in breast cancer, MDR1 can be induced by EVs through the activation of NFATc3 [181]. On the other hand, it has been demonstrated that p-STAT3 can be transferred to 5-fluorouracil-sensitive colorectal cancer cells to increase their resistance to 5-fluorouracil [182]. Furthermore, it has been observed that CLIC1 can be transferred to gastric cancer cells, thereby increasing levels of MDR1 and BCL2 and leading both to increased drug efflux and decreased apoptosis [183].

It is important to point out that other important molecular species, including both coding and non-coding RNAs, can also be transferred in EVs, which can also contribute enhanced cell resistance to various drug/compound treatments.

As platinum compounds are important components of the oncology arsenal, studies have been undertaken to assess transfer of resistance to these compounds. Often, it has been demonstrated that miRs influence resistance to platinum. For example, miR-19b influences resistance to platinum in colon cancer [184], while both miR-425-3p and miR-96 influence resistance to platinum in lung cancer cells [185,186]. Moreover, transfer of lncRNA HOTTIP increases resistance to platinum in gastric cancer cells, while increased serum HOTTIP lncRNA is associated with poor response to platinum [187]. Furthermore, coding RNAs are reported to influence sensitivity to platinum. For example, transfer of DNMT1 mRNA increases the resistance of ovarian cancer to platinum compounds [188].

Several other compounds are reported to be transferred through EVs as well For example, resistance to 5-fluorouracil in colon cancer cells is induced by both miR-145 and miR-34a [189], while the resistance of breast cancer cells to both adriamycin and tamoxifen are mediated by miR-222 transfer [190,191], and resistance of pancreatic cancer cells to gemcitabine is mediated by miR-155 transfer, leading to TP53INP1 modulation [192].

Interestingly, some pathways are more frequently targeted by some of the miRs, it has been reported that the PI3K/AKT pathway can be targeted by miR-21 in breast cancer cells [193] and by miR-1238 in glioblastoma cells [194].

## 8. EVs Used as Diagnostic Markers

EVs have been deemed as useful diagnostic markers in detecting the presence of a disease once the characteristics of malignancy are known. However, current methodologies for isolation and characterization of EVs are costly and not sufficiently standardized for de novo diagnostic protocols.

Nevertheless, one set of markers useful for diagnostics consists of fusion genes present in an assessed disease. These fusions occur more or less frequently depending on various malignancies, with hematologic malignancies, sarcomas, and prostate cancer presenting the most frequent fusion events [195]. For example, presence of *BCR-ABL* fusion genes in EVs, secreted by chronic myelogenous leukemia (CML) [18], in a patient’s plasma correlate with remission status in CML patients [196]. Although this approach cannot be directly transferred to a clinical diagnosis, as CML can be easily assessed in a patient’s blood, this can serve as an example for use in solid tumors, such as prostate cancer. The prostate cancer malignancy presents gene fusions in ~50% of cases, particularly of the *TMPRSS2–ERG* fusion gene as it is highly frequent [197]. Such an approach requires use of urine samples as isolated EVs present alterations in RNA signature(s) compared to those of control samples, including presence of the *TMPRSS2–ERG* fusion gene [198].

However, several common cancers do not present high frequencies of fusion genes. As a result, alternative strategies must be explored. For example, HER2-HER3 dimers from EVs have been assessed in HER2-positive breast cancer patients participating in a clinical trial (NCT04288141). Although the primary objective of this study was to identify a marker for resistance to anti-HER2 therapy, assessment of HER2-HER3 dimers from EVs may aid in identifying the tumor load in HER2-positive breast cancer patients (NCT04288141).

One of the most common alternative approaches under consideration for use of EVs as biomarkers is that of the dosage of the RNA species, particularly of miRs, determined by qRT-PCR followed by protein assessment, using either ELISA or mass spectrometry [199]. However, a major problem that may arise, particularly in assessing RNAs content in EVs, is that of sensitivity of RNA species to particular transport and storage conditions. Moreover, it has been observed that RNA assessment has rarely made it to a clinical setting, as these assessments have been generally constrained to viral loads, particularly of RNA viruses.

Thus, future studies should focus on either genetic or proteomic markers present in EVs, as these are more likely to be amenable for clinical implementation.

## 9. EVs Used in Anti-Cancer Therapy

**Figure 4:**
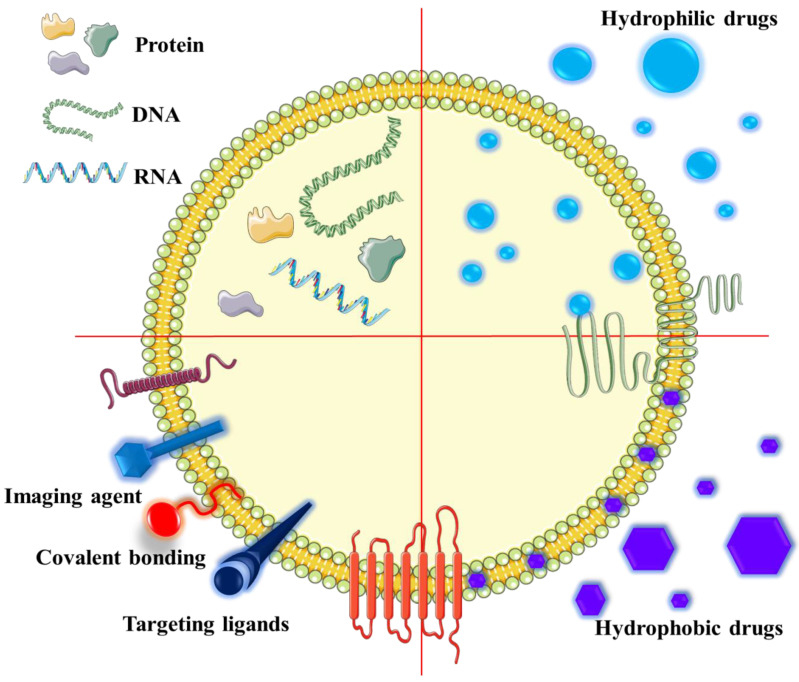
Properties of EVs useful in serving as drug delivery systems. These EVs consist of a lipid bilayer and an aqueous core, as they can incorporate hydrophilic drugs, hydrophobic drugs, nucleic acids (DNA, RNA), and proteins, as well as compounds (targeting ligands, covalent bonding, and imaging agents) that can be specifically attached to surfaces of EVs.

In recent years, accumulated knowledge of characteristics and cargos of EVs has suggested that these structures could serve as valuable biomarkers for diagnostic/prognosis, as well as therapeutic agents for treatment of various pathologies [200]. The emergence of EVs in cancer therapy serves as a valuable nanotechnology to overcome major worldwide cancer management problems [201]. Currently, there are many studies recommending use of EVs as delivery vectors for treatment of various cancer, following manipulation and engineering of these EVs to carry various molecules useful as therapeutic agents (Figure 4) [61,202,203].

Overall, use of EVs as delivery agents will aid in the transport of internal cargo via enhanced endocytosis, thus protecting the contents from degradation. In contrast to liposomes or other nanoparticles used as carriers, EVs can serve as ideal bioparticles for targeted therapies [204,205]. Interestingly, it is suggested that biodistribution of EVs is influenced by cell origin and characteristics, with cell-specific tropism, thereby highlighting their potential use in the field of precision medicine [206]. In this arena, studies have reported on the efficiency of EVs as biocompatible drug vectors, as well as exhibiting low cytotoxicity and immunogenicity, and demonstrating their internalizing capabilities within a cell, as well as crossing the blood–brain barrier [207,208]. These EVs are capable of encapsulating various molecules, such as siRNA, miR, and various chemotherapeutics [207]. For example, Ma et al. have demonstrated that EVs carrying anti-cancer compounds can be absorbed by regenerated tumor cells, thus offering opportunities for their use in overcoming acquired drug resistance during cancer therapy [208]. Furthermore, it is reported that EVs are more likely to be internalized under acidic conditions; therefore, tumor cells are preferentially targeted by EVs rather than cells from surrounding healthy tissues [209]. Moreover, paclitaxel-loaded EVs have been used to improve the efficiency of treatment in multidrug-resistant tumor cells [210]. Recently, it has been demonstrated that tumor-derived EVs exhibit tropism toward their parental tumor cells [211], wherein engineered EVs, derived from fibrosarcoma and cervical cancer cell lines encapsulating the drug Doxil, are monitored both in vivo and in vitro using either HT1080 or HeLa tumors/cell lines. As expected, mice treated with Doxil-encapsulated EVs have higher levels of Doxil at the tumor site than those treated with Doxil alone, thereby reducing nonspecific cytotoxic effects of this drug [211].

In another study on small cell lung cancer (SCLC), sFlt-1-enriched EVs (soluble fms-like tyrosine kinase-1) are reported to act as tumor suppressors in mice via suppression of angiogenesis and induction of apoptosis in SCLC tumor cells [212].

Furthermore, in vitro and in vivo experiments of colorectal cancer cells revealed that EVs carrying miR-128-3p enhanced sensitivities to oxaliplatin by targeting *Bmi1* and *MRP5* genes [213]. In another study, the inhibitory effects on cell proliferation and EMT of miR-34c were evaluated using EVs derived from mesenchymal stem cells for delivery of miR-34c into nasopharyngeal carcinoma cell lines, and increased sensitivity to radiotherapy was observed [214]. Moreover, EVs delivering miR-199a-3p successfully suppressed both invasion and proliferation of ovarian cancer cell lines [215].

Currently, numerous clinical trials are investigating potential uses of EVs for either diagnostic/prognostic purposes or for therapeutic treatments of cancer (Table 2). These clinical trials assessing the use of microvesicles underlines their critical roles in malignancies. For example, some of these ongoing studies are evaluating engineered EVs for use as therapeutics for the treatment of pancreatic cancer. While in a completed phase II clinical trial, a vaccine developed with tumor antigen-loaded dendritic cell-derived EVs for NSCLC patients responsive to induction chemotherapy have yielded promising results [216]. It is reported dendritic cell-derived EVs manufactured with IFN-γ serve as a viable immunotherapeutic for NSCLC patients [216]. Moreover, this construct boosts NKp30-dependent NK cell functions, but without adverse consequences on antigen-specific T cell responses when used as maintenance immunotherapy for these NSCLC patients [216].

All the abovementioned features of EVs render them as suitable candidates for targeted therapies, especially for cancer. However, there are some challenges in attempts for use in broad applications for cancer therapy, such as lack of standardized methods of isolation and purification of EVs, and challenges in identifying optimized methods for loading EVs with therapeutic compounds [217,218,219]. As of now, there are several studies on the use of engineered EVs loaded with different molecules/drugs for in vitro and/or in vivo experiments in cancer research, and these are summarized in Table 3.

## 10. Conclusions 

EVs represent particles released from both normal and malignant cells that have important biological roles in ensuring cell-to-cell communication, not only for neighboring cells but also for distant cells. EVs are classified as EVs, multivesicular bodies and apoptotic bodies, of different sizes, origin, and protein and lipid compositions. These EVs play critical roles in pathological states of cells, regulating all hallmarks of cancer cells and resistance to drug treatments, thus highlighting the potential of these entities in the management of cancer. EV capabilities in carrying different active biomolecules, such as different RNA species, DNA, and proteins for targeting recipient cells without triggering immune responses, have rendered them as valuable biological entities for use as therapeutic agents that can overcome the shortcomings of complex diseases, such as cancer.

## Figures and Tables

**Table 1:** Major characteristics of EVs.

Characteristics	Exosome	Multivesicular Body	Apoptotic Body	References
Size	Homologous 30–100 nm	Heterogenous 100–1000 nm	Heterogenous 1–5 μm	[31,32,33]
Origin	Multivesicular bodies fusion with cellular membrane	Direct outward budding or blebbing from the cellular membrane	Cellular membrane blebbing during cell death, cellular debris	[33,34]
Density	1.13–1.19 g/mL	1.25–1.30 g/mL	1.16–1.28 g/mL	[35]
Contents	Nucleic acids (DNA, mRNAs, miRs), lipids, specific proteins	Nucleic acids (DNA, mRNAs, miRs), lipids, specific proteins	Cellular organelles, cytosolic content (RNAs, fragmented DNA, proteins)	[33]
Protein components	Multivesicular body biogenesis (ALIX, TSG101), tetraspanins (CD9, CD63, CD81, CD82)	Death receptors (CD40 ligands), Cell adhesion (selectins, integrins)	Transcription and protein synthesis (histones)	[25,36]
Lipids	Lipidic molecules from the donor cells (include BMP)	Lipids from plasma membrane and resemble the donor cells (without BMP)	Characterized by phosphatidylserine externalization	[36,37]
Mechanism of release	Constitutive and/or cellular activation, depends on the cell type of origin	Cytoskeleton rearrangements, generation of membrane curvature, vesicle release, relocation of phospholipids to the outer membrane	Rho-associated kinase I and myosin ATPase activity	[37,38,39]
Determinant of controlled contents	The cellular origin and physiological state of the cell	No direct correlation	The cellular origin and stimuli	[35]
Markers	Membrane impermeable (PI negative), CD63, TSG101, Alix, flotillin, tetraspanins, HSP70, HSP90	Membrane impermeable (PI negative), selectin, integrin, flotillin-2, Annexin A1	Membrane permeable (PI positive), histone, DNA, Annexin V	[25,32]

MV, microvesicle; BMP, bone morphogenetic protein; PI, propidium iodide.

**Table 2:** Clinical studies exploring the use of EVs in cancer research studies.

Clinical Trial Identifier/Phase Status	Malignancy Investigated	EVs Use
NCT03236675/active, not recruiting	NSCLC	Detection of *EML4-ALK* fusion transcripts and T790M EGFR mutation
NCT03108677/recruiting	Osteosarcoma	Biomarkers for lung metastases, based on the RNS profile
NCT03985696/recruiting	Non-Hodgkin B-cell Lymphomas	Investigate EVs roles in immunotherapy, as carriers of therapeutic targets (CD20, PDL-1)
NCT03217266/recruiting	Soft tissue sarcoma	Detection of cell-free circulating tumor DNA mutations.
NCT02310451/unknown	Melanoma	Investigation of the effect of EVs produced by senescent melanoma cells
NCT03800121/recruiting	Sarcoma	Biomarkers for recurrence.
NCT03102268/unknown	Cholangiocarcinoma	Characterization of the ncRNAs in tumor derived EVs
NCT03911999/recruiting	Prostate cancer	Investigation of the relationship of urinary EVs and the aggressiveness of prostate cancer
NCT03711890/recruiting	Pancreatic cancer	Diagnostic biomarkers
NCT02869685/unknown	NSCLC	Detection of PD-L1 mRNA in plasma EVs
NCT03488134/active, not recruiting	Thyroid cancer	Urine exosomal proteins as biomarkers
NCT04258735/recruiting	Breast cancer	Diagnostic makers in a genomic panel
NCT02862470/active, not recruiting	Thyroid cancer	Urine EVs for the use as prognostic biomarkers
NCT01159288/completed	NSCLC	Treatment as tumor antigen-loaded dendritic cell-derived EVs
NCT04227886/recruiting	Rectal cancer	Biomarkers for toxicities and response to neoadjuvant therapy
NCT03608631/not yet recruiting	Pancreatic cancer	Treatment - mesenchymal stromal cells-derived EVs with KRAS G12D siRNA
NCT01779583/unknown	Gastric cancer	Prognostic and predictive biomarkers
NCT03874559/recruiting	Rectal cancer	Diagnostic biomarkers

Abbreviations: EVs- Extracellular vesicles; ncRNA–non-coding RNA; NSCLC–non-small cell lung cancer; PDL-1- programmed cell death ligand 1; siRNA–silence interfering RNA.

**Table 3:** Studies focused on investigating the effect of EVs-based therapy in in vivo and in vitro.

Pathology	EVs/Extracellular Vesicles Derived From	Cargo	Method of Engineering	In Vitro/In Vivo	Effect	Reference
Ovarian cancer	Fibroblasts from normal omentum	miR-199a-3p	Electroporation	SKOV3ip1, OVCAR3, CaOV3 and SKOV3-13	Inhibition of ovarian cancer cell proliferation, invasiveness, and c-Met expression.	[215]
				BALB/c nude mice	Inhibition of ovarian cancer peritoneal dissemination.	
Cancer	M1 macrophages	aCD47 and SIRPα	Polarization and conjugation	4T1tumor-bearing BALB/c mice	enhanced the phagocytosis of macrophages	[220]
Cancer	Bel7402 cell line	Doxorubicin-loaded PSiNPs (porous silicone nanoparticles)	Incubation	BALB/c mice and C57BL/6 mice bearing H22 tumors	Enhanced tumor accumulation of doxorubicin	[221]
Small cell lung cancer	BEAS-2B and NCI-H69 cell lines	sFlt-1	Cloning sFlt-1 into a lentivirus and obtaining engineered cell lines overexpressing sFlt-1	Nude mice with NCI-H69 xenografts	Induction of tumor apoptosis and inhibition tumor cell proliferation.	[212]
Glioma	RAW264.7 cells	Doxorubicin	Incubation	GL261 cells and RAW264.7 cells	Uptake of loaded EVs is higher in cancer cells than in normal cells	[222]
				C57BL/6 mice	Increased blood circulation time	
Breast cancer	Artificial chimeric EVs (ACEs)	Doxorubicin	Integration of RBCs and MCF-7 cell membrane proteins into synthetic phospholipid bilayers.	MCF-7 cells	Inhibition of cellular growth	[223]
				BALB/c nude mice and ICR mice	Doxorubicin accumulation in tumor improving anti-tumor efficacy	
Hepatocellular carcinoma	Plasma of healthy blood donors	miR-31 and miR-451	Electroporation	HepG2 cells	Increased cancer cell apoptosis.	[224]
Breast cancer	MSC	Doxorubicin	Electroporation	BT-474 and MDA-MB231 cells	Reduced cell viability, but with no significant differences between free DOX and EVs encapsulate DOX	[225]
Her2+ Breast Cancer	HEK 293T cells	siRNA	pLEX-LAMP-DARPin lentiviral transduction in HEK 293T cells	SKBR3 cells	Increased suppression of target gene (*TPD52*) compared to untreated cells and negative control (unloaded EVs)	[226]
Breast cancer	MSC	miR-379	lentiviral transduction of MSCs	BALB/c nude mice	Reduction in tumor size compared to the negative control (NTC extracellular vesicles)	[227]
NSCLC	RAW 264.7 cells	Paclitaxel	Sonication and incubation (including vectorization of EVs-AA-PEG-exoPTX)	C57BL/6 mice with established mCherry-3LL-M27 metastases	Stronger suppression of metastases growth and greater survival time as compared to Taxol, or non-vectorized exoPTX formulation	[228]
Pancreatic cancer	Normal fibroblast-like mesenchymal cells	siRNA or shRNA targeting Kras^G12D^	Electroporation	Panc-1 cells	Enhanced apoptosis and decreased proliferation	[229]
				Nu/nu mice with orthotopic Panc-1 tumors	Controlled growth of tumors	
Chronic myeloid leukemia	HEK293T cells	Imatinib (IL3 EVs)	Incubation	LAMA84 and K562R cells	Reduction in cell viability compared to empty imatinib loaded EVs	[230]
				NOD/SCID mice	Reduction in tumor size	
Melanoma	B16BL6 cells	CpG-DNA (SAV-LA EVs)	Incubation	C57BL/6J mice and BALB/c nu/nu mice	Inhibition of tumor growth.	[231]
Breast cancer	immature mouse dendritic cell line (imDC)	Doxorubicin (iRGD-positive EVs)	Electroporation	MDA-MB-231	Inhibition of cell proliferation	[232]
				MDA-MB-231 tumor-bearing BALB/c nude mice	Inhibition of tumor growth due to effective accumulation of Dox at tumor sites	
Breast cancer	HEK293	let-7 (GE11-positive EVs)	lipofection	RAG2–/– mice	Suppression of tumor growth	[233]

Abbreviations: EVs, extracellular vesicles; MSC, mesenchymal stem cells; NSCLC, non-small cell lung cancer; siRNA, small interfering RNA; shRNA, short hairpin RNA; DC; dendritic cells.
